# Thrombus Density in Acute Basilar Artery Occlusion Depends on Slice Thickness and the Method of Manual Thrombus Delineation

**DOI:** 10.3390/life12081273

**Published:** 2022-08-19

**Authors:** Liang Shu, Johannes Meyne, Olav Jansen, Ulf Jensen-Kondering

**Affiliations:** 1 Department of Radiology and Neuroradiology, University Hospital Schleswig-Holstein, Campus Kiel, 24105 Kiel, Germany; 2 Department of Neurology, Shanghai Ninth People’s Hospital, Shanghai Jiao Tong University School of Medicine, Shanghai 200025, China; 3 Department of Neurology, University Hospital Schleswig-Holstein, Campus Kiel, 24105 Kiel, Germany; 4 Department of Neuroradiology, University Hospital Schleswig-Holstein, Campus Lübeck, 23538 Lübeck, Germany

**Keywords:** basilar artery occlusion, thrombus, density, CT, hyperdense artery sign

## Abstract

*Introduction*: High thrombus attenuation on CT has been suggested as a predictor of successful recanalization. It is as well speculated that thrombi of different density may be susceptible to different methods of mechanical thrombectomy. In this study we sought to determine the effect of different methods of manual thrombus delineation and reconstructed slice thickness on thrombus density. *Material and Methods*: Fifty-six patients with acute occlusion of the basilar artery treated with endovascular therapy were retrospectively included. Clinical, demographic, radiological and outcome parameters were collected. Two raters measured absolute and relative thrombus density employing three different methods (one region of interest, three regions of interest, whole thrombus delineation) and using three different reconstructed slice thicknesses (0.625, 2.5 and 5 mm) of the original admission CT. *Results*: Thirty-nine patients were successfully recanalized (thrombolysis in cerebral infarction score ≥ 2b). Good clinical outcome (modified Rankin scale ≤ 2) occurred significantly more often in the recanalized group (36 vs. 6%, *p* = 0.023, Fisher’s exact test), in the non-recanalized group symptomatic intracranial hemorrhage occurred more often (9 vs. 29%, *p* = 0.001, Fisher’s exact test). Absolute and relative thrombus density were largely different between methods and slice thicknesses. Multiple regression showed a decrease of thrombus density with increasing slice thickness (β = −3.98, *p* < 0.001) and logistic regression showed a statistically significant but very small relation between density and recanalization (β = 0.006, odds ratio (95% confidence interval) = 1.006 (1.003–1.01), *p* < 0.001). *Conclusions*: The methods for manual thrombus delineation and reconstructed slice thickness had a significant influence on absolute and relative thrombus density. Density alone may be of limited value as a predictive marker for recanalization success in acute occlusion of the basilar artery. Standards for density measurements must be defined when comparing different studies and when evaluating different methods of mechanical thrombectomy.

## 1. Introduction

Acute occlusion of the basilar artery (BAO) is a rare condition but is associated with a very high level of mortality and bad functional outcome [1,2,3]. Restoration of blood flow in the basilar artery is the primary goal of therapeutic approaches. Both systemic and endovascular approaches are being performed with non-randomized trials indicating an advantage for endovascular approaches [4] but randomized-controlled data suggest non-inferiority of intravenous thrombolysis in a subset of patients [5]. High thrombus attenuation on non-enhanced cranial CT (NECCT) has been suggested as a predictor of successful recanalization for both intravenous thrombolysis and endovascular thrombectomy [6,7], but this is not undisputed.

However, the results of the different studies are not directly comparable because different methods of thrombus density measurements have been used which yield different density results on the same NECCT [8]. Further, slice thickness has been shown to influence thrombus density. No standard slice thickness on NECCT has been agreed upon and many different slice thicknesses are used in the literature to measure thrombus density.

In this study, we sought to determine the effect of different methods of manual thrombus delineation and different reconstructed slice thicknesses on thrombus density. As a secondary analysis, we compared yielded thrombus density in successfully recanalized and non-recanalized patients.

## 2. Materials and Methods

### 2.1. Patients, Clinical and Demographic Data

The study was approved by the local ethics committee (Ethikkommission der Medizinischen Fakultät der Christian-Albrechts-Universität zu Kiel). Patients consented to the retrospective use of acquired data at hospital admission. If consent for usage was not given, only anonymized data were used. Fifty-six consecutive patients with BAO treated with endovascular therapy (EVT) at our institution between 2007 and 2016 were retrospectively included. Inclusion criteria were the following: presence of an NECCT performed at our institution and a complete set of clinical information including pre-interventional National Institute of Health Stroke Score (NIHSS) assessed by the admitting stroke neurologist, 90-day modified Rankin Scale (mRS) assessed either by face-to-face consultation or by telephone interview, onset-to-treatment time and stroke etiology after clinical work-up including follow-up brain imaging (CT or MRI), cervical vessel assessment (Doppler ultrasound, CTA or MRA), electrocardiogram, laboratory tests and transthoracic or transesophageal echocardiography, as clinically feasible. Additionally, age and sex were extracted.

### 2.2. Imaging and Image Reconstruction

Pre-interventional NECCT was performed on a Philips Brilliance 64 (120 kV, 320 mAs). NECCTs were reconstructed to 2.5 and 5 mm slice thickness using the primary slice thickness of 0.625 mm. In 19 patients, the primary slice thickness was not available, resulting in a total of 149 NECCTs, i.e., 37 NECCTs with 0.625 mm slice thickness and 56 NECCTs with 2.5 and 5 mm each.

A CT angiography (80 kV, 280 mAs, 80 mL bolus of 350 mg I/mL, followed by 40 mL saline flush both injections at 5 mL/s, image acquisition from the aortic arch to the vertex) performed at the time of the NECCT was available in 51 patients.

### 2.3. Thrombus and Occlusion Length

Thrombus length on NECCT was calculated as previously described [9]. In short, a small region of interest (ROI) was chosen in the course of a hyperdense vessel. Pixels between 55 and 80 Hounsfield units (HU) served as a seed for region-growing into adjacent pixels between 40 and 80 HU. Thrombus length was then defined along the middle axis of this volume.

Occlusion length, defined as an opacification gap on CT angiography, was determined by one author (board-certified stroke neurologist) and verified by another (board-certified radiologist), both blinded to the results of thrombus length measurement on NECCT.

### 2.4. Occlusion Site and Symptomatic Intracranial Hemorrhage

Further, the site of occlusion of the basilar artery (proximal, mid, top), and the occurrence of symptomatic intracranial hemorrhage (according to ECASS II criteria) on follow-up brain imaging (CT or MRI) were recorded.

### 2.5. Qualitative Assessment

The presence of a hyperdense basilar artery sign (HDBAS) was rated by two independent raters (board-certified stroke neurologist and board-certified radiologist) on all NECCTs. Disagreement was solved by consensus.

### 2.6. Quantitative Assessment—Absolute and Relative Thrombus Density

**Figure 1:**
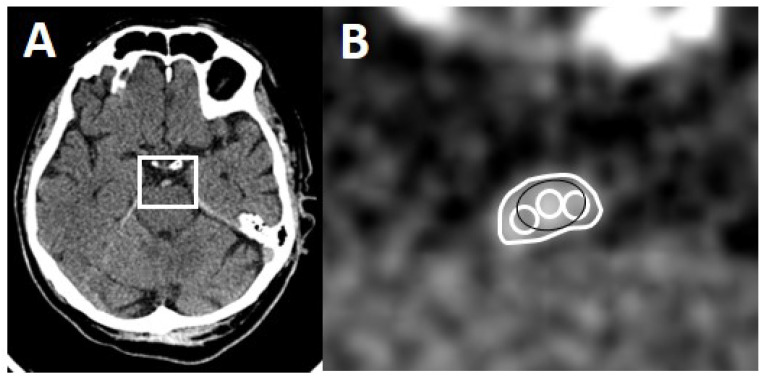
Methods of thrombus delineation. (**A**) Axial CT section with a hyperdense basilar artery. (**B**) Magnified region of interest (white box) with the three methods of thrombus delineation. Method 1: black RoI, Method 2: three white circles, Method 3: large white RoI.

One rater (board-certified stroke neurologist) blinded to clinical and imaging results measured absolute thrombus density in all 56 patients on all NECCTs with all three available slice thicknesses (0.625, 2.5 and 5 mm). Three different methods were used. Method 1: one round or oval region of interest (RoI) was drawn onto the most hyperdense part of the thrombus [10]. Method 2: three small (or two if too small) oval or circular RoIs were drawn onto the thrombus [7]. Method 3: the outline of the whole thrombus was delineated by carefully excluding pixels outside the thrombus as a RoI on each slice of the NECCT [11]. For Method 2 and 3, the sum of the obtained density values was then divided by the number of drawn RoIs. Densities > 100 HU were considered calcifications and were excluded (Figure 1). 

Additionally, this first rater calculated relative thrombus density by measuring density in the patent part of the basilar artery (or the proximal vertebral or posterior cerebral artery as anatomically adequate) using the same three methods as described above and subsequently dividing the obtained values by absolute thrombus density. This resulted in a total of 894 density measurements derived from 2604 unique datapoints on 149 NECCTs.

A second rater (board-certified radiologist) blinded to the results of the first rater measured absolute and relative thrombus density in 20 patients to calculate inter-rater agreement. Henceforth, only the results of the first rater were used for subsequent calculations.

### 2.7. Treatment Modality and Recanalization Success

Treatment was initiated at the discretion of the admitting neurologist and neurointerventionalist according to national and international guidelines. Recanalization was rated using the thrombolysis in cerebral infarction (TICI) score. Recanalization success was categorized into recanalized (TICI ≥ 2b) and non-recanalized (TICI < 2b) by the neurointerventionalist who performed the thrombectomy. Treatment strategy (intravenous and/or intraarterial recombinant tissue plasminogen activator, devices used for mechanical thrombectomy) was recorded.

### 2.8. Statistics

Continuous clinical and imaging parameters were subjected to the Shapiro–Wilks test to detect normal distribution. These parameters were compared between the two groups with the adequate statistical test (χ^2−^ or Fisher’s exact test for categorical variables, unpaired *t*-test for normally distributed continuous variables and Mann–Whitney test for non-normally distributed continuous variables).

Agreement between raters for the HDBAS was calculated using Cohen’s κ. The frequency of the HDBAS was compared between NECCT with different slice thicknesses using the G test with *p*-values adjusted by the FDR method (Benjamini–Hochberg) for multiple comparisons. Agreement between raters for absolute and relative thrombus and vessel density was calculated using the ICC.

Normal vessel density, absolute and relative vessel density were displayed as box plots and compared using ANOVA and the Tukey–Kramer post-hoc test. Absolute and relative thrombus density between patients with (TICI ≥ 2b) and without (TICI < 2b) recanalization were compared with an unpaired two-sided t-test with *p*-level adjustment for multiple comparisons (after Bonferroni correction, *p* = 0.0055). The Bland–Altman plot was used to calculate and graphically demonstrate the median mean differences between applied methods and slice thickness.

The relation between slice thickness and the method of thrombus delineation on thrombus density was evaluated using multiple linear regression. Logistic regression was performed to evaluate the influence of thrombus length and density on recanalization.

R (version 3.5.0, R Foundation for Statistical Computing, Vienna, Austria) and StatView (Version 5.0.1, SAS Institute Inc., Cary, NC, USA) were used for statistical calculations. A *p*-value of <0.05 was considered statistically significant.

## 3. Results

### 3.1. Clinical and Imaging Parameters

Demographic, imaging and clinical information of the included patients are listed in Table 1. Fifty-one of these patients were included in a previous study on BAO [10]. Thirty-nine patients were classified as recanalized (TICI ≥ 2b), seventeen patients as non-recanalized (TICI < 2b).

### 3.2. Qualitative Assessment

The HDBAS was present in 92% (*n* = 34, κ = 0.78) of 0.625 mm slice thickness, in 73% (*n* = 41, κ = 0.75) of 2.5 mm slice thickness and 71% (*n* = 40, κ = 0.54) of 5 mm slice thickness. Slice thickness and the presence of a hyperdense artery sign were significantly associated (*p* = 0.047). The post-hoc test revealed a significant difference between 0.625 mm and 5 mm (*p* = 0.029), 0.625 mm and 2.5 mm (*p* = 0.029) but not between 2.5 and 5 mm slice thickness (*p* = 0.833).

### 3.3. Quantitative Assessment—Absolute and Relative Thrombus Density

Agreement between raters for absolute and relative density measurements was good (ICC = 0.84).

**Figure 2:**
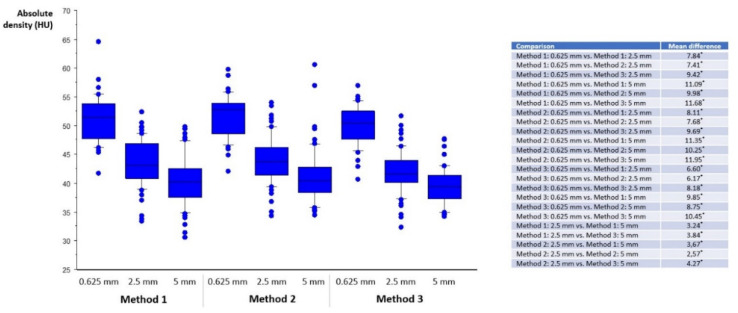
Normal vessel density. On the x-axis, slice thickness (0.625, 2.5, 5 mm) and the method of density measurements are listed (left three bars: Method 1; middle three bars: Method 2; right three bars: Method 3). For visual clarity, statistical significance (*, *p* < 0.05) is listed in the right-hand table. Median, 25th, and 75th percentile are displayed as boxplots, 10th and 90th as whiskers, dots represent outliers.

**Figure 3:**
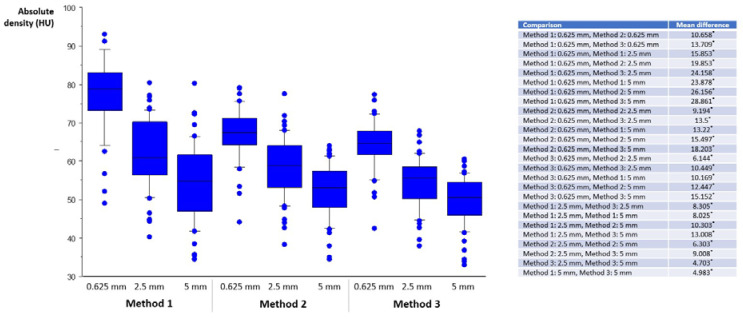
Absolute thrombus density. On the *x*-axis, slice thickness (0.625, 2.5, 5 mm) and the method of density measurements are listed (left three bars: Method 1; middle three bars: Method 2; right three bars: Method 3). For visual clarity, statistical significance (*, *p* < 0.05) is listed in the right-hand table. Median, 25th, and 75th percentile are displayed as boxplots, 10th and 90th as whiskers, dots represent outliers.

**Figure 4:**
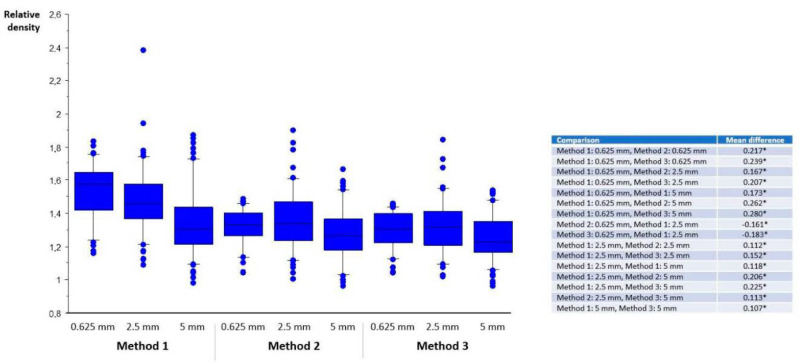
Relative thrombus density. On the *x*-axis, slice thickness (0.625, 2.5, 5 mm) and the method of density measurements are listed (left three bars: Method 1; middle three bars: Method 2; right three bars: Method 3). For visual clarity, statistical significance (*, *p* < 0.05) is listed in the right-hand table. Median, 25th, and 75th percentile are displayed as boxplots, 10th and 90th as whiskers, dots represent outliers.

**Figure 5:**
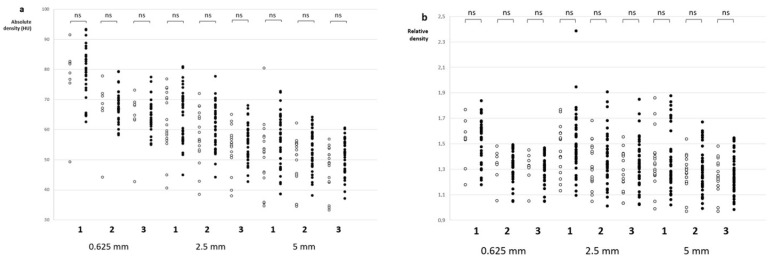
Comparison of absolute (**a**) and relative (**b**) thrombus density values in patients with TICI 200 < 2b (empty circles) and TICI ≥ 2b (solid circles). (ns: not significant, unpaired *t*-test).

Normal vessel density, absolute and relative thrombus density are displayed in Figure 2, Figure 3 and Figure 4. Absolute and relative thrombus density values were largely and significantly different between methods and between reconstructed slice thicknesses, and ranged from 49.59 ± 6.55 HU (Method 3, slice thickness 5 mm) to 78.45 ± 9.44 HU (Method 1, slice thickness 0.625 mm). However, no statistically significant difference was found when comparing recanalized and non-recanalized patients (Figure 5). Bland–Altmann plots showed a mean difference of up to 21.97 HU with limits of agreement of ±15.04 HU (Method 1, slice thickness 0.625 vs. 5 mm) for absolute thrombus density and 10.76 HU with limits of agreement of ±10.02 HU (Method 1, slice thickness 0.625 vs. 5 mm) for normal vessel density (Supplemental Figure S1).

Multiple regression showed a statistically significant decrease in thrombus density with increasing slice thickness (β = −3.98, *p* < 0.001). Method 1 yielded a greater thrombus density when compared to method 2 (β = 5.10, *p* < 0.001), method 2 yielded greater thrombus density when compared to method 3 (β = 3.44, *p* < 0.001) and method 1 yielded a greater thrombus density when compared to method 3 (β = 8.54, *p* < 0.001). Logistic regression showed no relation between thrombus length and recanalization (β = 0.007, *p* = 0.151) but a statistically significant but very small relation between density and recanalization (β = 0.006, OR (95%CI) = 1.006 (1.003–1.01), *p* < 0.001).

## 4. Discussion

We can report two main results of our cohort of patients with acute occlusion of the basilar artery. First, thrombus density measured with different manual methods on different reconstructed slice thicknesses yielded very different results. As an overall trend, we found increased density with decreased slice thickness. An increased presence of the hyperdense basilar artery sign on thin slice NECCT corroborates this finding. Further, placing one RoI yielded the highest density whereas encircling the whole thrombus yielded the lowest density values. Second, thrombus density was a predictor for successful recanalization but demonstrated only a very weak relation (β = 0.006).

High attenuation is observed in erythrocyte-rich “red” thrombi due to their high cellularity in contrast to fibrin-dominant “white” thrombi. Several experimental [12,13] and clinical imaging [14] pathological correlation studies support that notion. It was repeatedly stated that successful recanalization is more common with high attenuating thrombi [6,7,10,11,15].

However, caution must be exerted. The observation that high attenuating thrombi are more likely to be recanalized is not generally made. A growing number of studies (for a summary of the key references, see Supplemental Table S1) draw into question the relationship between high thrombus density and recanalization success [16,17,18,19,20,21,22] for which several factors may be responsible.

**Figure 6:**
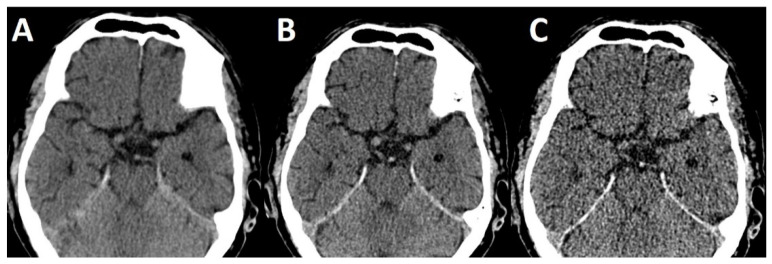
NECCT with 5 mm (**A**), 2.5 mm (**B**) and 0.625 mm (**C**) reconstructed slice thickness. Note the absence of a hyperdense basilar artery on 5 mm (**A**) slice thickness.

First, slice thickness on NECCT is pivotal when quantitative measurements of thrombus density are made [9]. In the literature, a wide range of slice thicknesses (0.625 mm [9], 1 mm [22], 2.5 mm [10], 3 mm [6], 4.8 mm [7] and 5 mm [20]) were employed. In this study, an increase of 1 mm slice thickness led to a decrease of approximately 4 HU in thrombus density. The decreasing rate of a hyperdense artery sign on thick slice reconstruction can be readily explained by the partial volume effect [9]. Especially short thrombi will be lost to blurring with surrounding cerebrospinal fluid when slice thickness exceeds thrombus dimensions (Figure 6). Kirchhoff and co-workers reported an additional influence of tube current with increasing thrombus density under increasing tube current [23] which could be the target for studies using dual-energy [12,24] or future spectral CT imaging studies.

Second, several different methods to measure thrombus density were used across studies [6,7,10], which might explain some of the discrepant outcomes. In this study, multiple regression analysis revealed a difference of up to 8 HU between methods.

Third, the overwhelming majority of studies were conducted in the anterior circulation and there are few studies reporting on thrombus density and recanalization success in the posterior circulation [25,26,27]. One of the reasons may be that the basilar artery is prone to beam hardening artifacts in the posterior fossa [28] which are not present in the anterior circulation.

Fourth, different methods of mechanical thrombectomy may be effective in different types of thrombi of different compositions and densities [22,29]. It was reported that the number of passes [30], procedure time [30,31] and distal emboli [31] differed depending on the density of the thrombus (but not in this study, data not shown). As tissue classification predicts infarct volume and clinical outcome [32], thrombus density measurement and other thrombus property assessments [33] may lead to stratification and choice of the appropriate mechanical thrombectomy device and/or strategy and should be the focus of further studies.

Fifth, the correlation between thrombus density and thrombus composition may be more complex and multifactorial, including other factors such as local stasis and collateral circulation [34,35] which might influence thrombus composition in its distal and proximal end in opposite directions. None of the methods utilized hitherto took into account heterogenous thrombi.

## 5. Conclusions

The utilized methods for manual thrombus delineation yielded largely different results and reconstructed slice thickness had a significant influence on absolute and relative thrombus density, which complicates comparisons between studies. Standards, preferably with thin sliced (<5 mm) reconstructions, are needed. However, the different methods and reconstructed slice thickness did only badly predict recanalized and non-recanalized patients. Density alone may be of limited value as a prognostic marker for recanalization success in acute occlusion of the basilar artery but may be helpful in choosing an appropriate endovascular treatment strategy pending further research. Standards for density measurements are needed when comparing different studies and when evaluating different methods of mechanical thrombectomy.

In the future, automatic thrombus detection and measurement [36] and machine learning algorithms [37] may aid the human reader. Recently, Qazi and co-workers presented a method to perform a complete 3D model of the thrombus, which might also be implemented into clinical routines [38].

## Figures and Tables

**Table 1:** Demographic, clinical and radiological characteristics.

Characteristics	All Patients (*n* = 56)	TICI ≥ 2b (*n* = 39)	TICI < 2b (*n* = 17)	*p*-Value
Age, mean (SD) years	72 (24–90)	69 (11)	72 (12)	0.404 *
Female sex (%)	18 (29)	14 (36)	4 (24)	0.505 ^†^
Baseline NIHSS, median (range)	17 (0–32)	16 (2–32)	18 (0–30)	0.181 ^§^
Onset to treatment, median (range) min	222 (65–3290)	223 (79–3290)	205 (65–2625)	0.964 ^§^
Etiology				
Cardioembolic (%)	31 (55)	20 (51)	11 (65)	0.353 ^†^
Atherosclerotic (%)	16 (29)	13 (33)	3 (18)	0.338 ^#^
Other confirmed (%)	6 (9)	4 (10)	2 (12)	1 ^#^
Unknown (%)	3 (5)	2 (5)	1 (6)	1 ^#^
Good clinical outcome, mRS ≤ 2 (%)	15 (27)	14 (36)	1 (6)	**0.023** ^#^
Occlusion length CTA, median (range) mm	10.9 (2.5–65.9) *^n^* ^= 51^	11.9 (2.5–65.9) *^n^* ^= 37^	10.85 (4.5–45.6) *^n^* ^= 14^	0.966 ^§^
Thrombus length NECCT, median (range) mm	8.4 (2.5–63.6)	8.9 (2.5–63.6)	7.5 (2.5–33.4)	0.610 ^§^
Site of occlusion				
Proximal (%)	11 (20)	5 (13)	6 (35)	0.071 ^#^
Mid (%)	23 (41)	19 (49)	4 (24)	0.078 ^†^
Top (%)	22 (39)	15 (38)	7 (41)	0.489 ^†^
SICH (%)	5 (9)	0 (0)	5 (29)	**0.001** ^#^
Intervention strategy				
IA rtPA (%)	5 (9)	1 (2)	4 (24)	**0.034** ^#^
Mechanical (any) + IA rtPA (%)	16 (29)	11 (28)	5 (29)	1 ^#^
Mechanical (any) only (%)	35 (63)	27 (69)	8 (47)	0.115 ^†^
Mechanical including stent retrievers (%)	36 (64)	28 (72)	8 (47)	0.075 ^†^
IV rtPA (%)	9 (16)	7 (18)	2 (12)	0.562 ^†^

*: *t*-test, ^†^: Chi-square test, ^§^: Mann–Whitney U-test, ^#^: Fisher’s exact test. TICI: thrombolysis in cerebral infarction, SD: standard deviation, NIHSS: National Institute of Health Stroke Score, mRS: modified Rankin scale, CTA: computed tomography angiography, NECCT: non enhanced cranial computed tomography, SICH: symptomatic intracranial haemorrhage, IA: intraarterial, rtPA: recombinant tissue plasminogen activator, IV: intravenous.

## Data Availability

All relevant data is available within the manuscript.

## Supplementary Materials

The following supporting information can be downloaded at: https://www.mdpi.com/article/10.3390/life12081273/s1, Figure S1: Bland-Altmann plots; Table S1: Summary of the key references cited in the manuscript in chronological order.
