# Supplementary material for: Thrombus Density in Acute Basilar Artery Occlusion Depends on Slice Thickness and the Method of Manual Thrombus Delineation
Source: Life (Basel). 2022 Aug 19;12(8):1273. doi: 10.3390/life12081273 (PMC9409736; doi:10.3390/life12081273)
Supplement: Supplementary file 1 [file life-12-01273-s001.zip › life-1815292-supplementary.pdf]

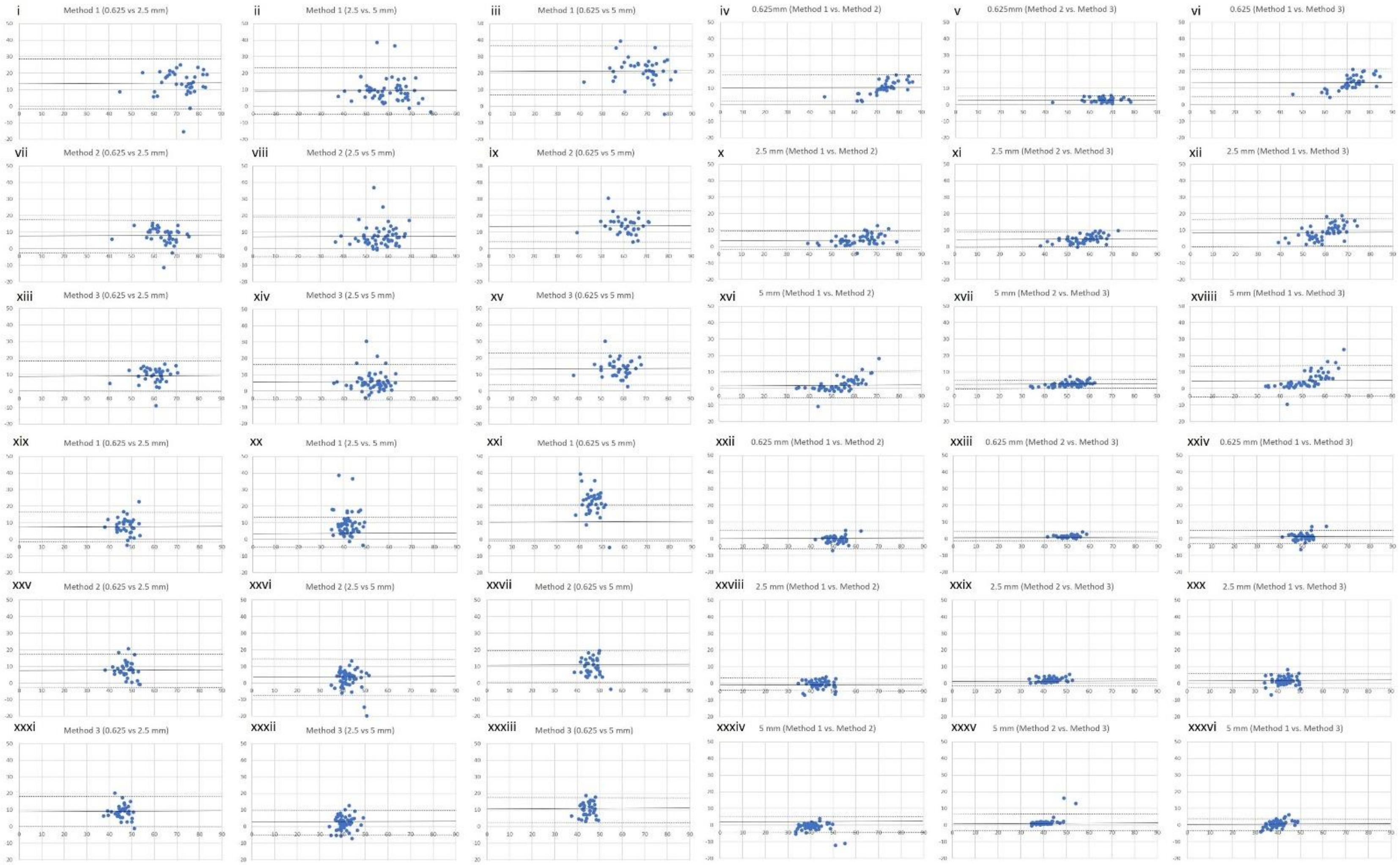

Supplemental Figure S1. Bland-Altmann plots visualize the varying differences between the different methods of manual thrombus delineation and utilized slice thickness. (Normal vessel i–xviii, thrombus xix–xvi, solid line: mean difference, dotted lines: upper and lower limits of agreement).

Supplemental Table S1. Summary of the key references cited in the manuscript in chronological order.

Study	n	Treatment	Site of Occlusion	Slice Thickness	Method	Absolute Density (HU)	Relative Density (rHU)	Correlation with	
								Recanalization	Outcome
Puig et al., 2012	87	IVT	AC	3 mm	Whole	42 vs. 50	1.52 vs. 1.15	Yes	NA
Froehler et al. 2013	67	EVT	AC/BA (n=4)	4.8 mm	3 RoIs	NA	1.46[a] vs. 1.03[b]	Yes	NA
Moftakhar et al., 2013	90	IVT<br>EVT<br>EVT+IVT	AC and PC	2.5 mm	1 RoI	55±24.8 vs. 52±14.2<br>55±24.5 vs. 45±19.1<br>51±22.3 vs. 45±13	1.58 vs. 1.39<br>1.66 vs. 1.40<br>1.7 vs. 1.3	Yes	NA
Yilmaz et al., 2013	70	EVT	AC	5 mm	1 RoI	50.1 vs. 48.7	NA	No	NA
Spiotta et al., 2014	141	EVT	NA	4.8 mm	3 RoIs	57.1±16.3 vs 68.7±43.2	NA	No	No
Topcuoglu et al., 2014	105	IVT<br>IA rtPA	AC	5 mm	1 RoI	47.4 vs. 49.4	1.13 vs. 1.17	NA	No
Mokin et al., 2015	41	EVT	AC	5 mm	Whole	49.9±7.6 vs. 43.8±6.6	1.2±0.2 vs. 1.0±0.1	Yes	NA
Angermaier et al., 2015	171	EVT	AC	4.5 mm	NA	NA	NA	No	NA
Jagani et al., 2017	118	EVT	AC/BA (n=19)	5 mm	1 RoI	50.1±7.4 vs. 53±12.7	1.31±0.22 vs. 1.37±0.34	No	NA
Shu et al. 2017	51	EVT	BA	2.5 mm	Whole	52.3±5.8 vs 48.4±6.7	1.29±0.14 vs 1.35±0.15	No	Yes
Ye et al., 2019	64	EVT	AC	1 mm	1 RoI	65.27 vs. 62.19	NA	No	NA
Bruggeman et al., 2022	566	EVT	AC	<2.5 mm	3 RoIs	NA	NA	No	No

(IVT: i.v. thrombolysis, EVT: endovascular therapy, AC: anterior circulation, PC: posterior circulation, BA: basilar artery, NA: not applicable). a: hyperdense, b: isodense.
